# Development and Validation of Prediction Formula of Wingate Test Peak Power From Force–Velocity Test in Male Soccer Players

**DOI:** 10.3389/fpsyg.2021.729247

**Published:** 2021-11-29

**Authors:** Pantelis T. Nikolaidis, Beat Knechtle

**Affiliations:** ^1^School of Health and Caring Sciences, University of West Attica, Athens, Greece; ^2^Exercise Physiology Laboratory, Nikaia, Greece; ^3^Institute of Primary Care, University of Zurich, Zurich, Switzerland

**Keywords:** all-out test, anaerobic power, cycle ergometer, football, muscle strength, performance, speed

## Abstract

Peak power of the Wingate anaerobic test (WAnT), either in W (Ppeak) or in W.kg^–1^ (rPpeak), has been widely used to evaluate the performance of soccer players; however, its relationship with force–velocity (F-v) test (e.g., whether these tests can be used interchangeably) has received little scientific attention so far. The aim of this work was to develop and validate a prediction equation of Ppeak and rPpeak from F-v characteristics in male soccer players. Participants were 158 adult male soccer players (sport experience 11.4 ± 4.5 years, mean ± standard deviation, approximately five weekly training units, age 22.6 ± 3.9 years, body mass 74.8 ± 7.8 kg, and height 178.3 ± 7.8 cm) who performed both WAnT and F-v test. An experimental (EXP, *n* = 79) and a control group (CON, *n* = 79) were used for development and validation, respectively, of the prediction equation of Ppeak and rPpeak from F-v test. In EXP, Ppeak correlated very largely with body mass (*r* = 0.787), fat-free mass (*r* = 0.765), largely with maximal power of F-v test (*P*_*max*_; *r* = 0.639), body mass index (*r* = 0.603), height (*r* = 0.558), moderately with theoretical maximal force (*F*_0_; *r* = 0.481), percentage of body fat (*r* = 0.471), fat mass (*r* = 0.443, *p* < 0.001); rPpeak correlated with *rP*max (largely; *r* = 0.596, *p* < 0.001), theoretical maximal velocity (*v*_0_; moderately; *r* = 0.341, *p* = 0.002), *F*_0_ (small magnitude; *r* = 0.280, *p* = 0.012), BF (*r* = −0.230, *p* = 0.042), and fat mass (*r* = −0.242, *p* = 0.032). Ppeak in EXP could be predicted using the formula “44.251 + 7.431 × body mass (kg) + 0.576 × *P*_*max*_ (W) – 19.512 × F_0_” (*R* = 0.912, *R*^2^ = 0.833, standard error of estimate (SEE) = 42.616), and rPpeak from “3.148 + 0.218 × *rP*max (W.kg^–1^) + v0 (rpm)” (*R* = 0.765, *R*^2^ = 0.585, SEE = 0.514). Applying these formulas in CON, no bias was observed between the actual and the predicted Ppeak (mean difference 2.5 ± 49.8 W; 95% CI, −8.7, 13.6; *p* = 0.661) and rPpeak (mean difference 0.05 ± 0.71 W.kg^–1^; 95% CI, −0.11, 0.21, *p* = 0.525). These findings provided indirect estimates of Ppeak of the WAnT, especially useful in periods when this test should not be applied considering the fatigue it causes; in this context, the F-v test can be considered as an alternative of exercise testing for estimating the average Ppeak of a group of soccer players rather than for predicting individual scores when the interindividual variation of performance is small.

## Introduction

Performance in soccer has been shown to rely on movements such as sprinting, passing, shooting, jumping, and change of direction (Lepschy et al., 2021; Longo et al., 2021). Considering the short duration and maximal effort characterizing these movements, it was not surprising that the Wingate anaerobic test (WAnT), an all-out 30-s test on a cycle ergometer, was widely used to evaluate performance in this team sport (Chtourou et al., 2019; Bahenský et al., 2020). The most popular index of the WAnT has been the peak power expressed either in absolute (Ppeak) or relative to body mass values (rPpeak). Ppeak was related largely with linear and non-linear sprint performance and moderately with 20-m sprint performance, and could differentiate soccer players among playing positions (Joo and Seo, 2016; Nikolaidis et al., 2016; Almansba et al., 2019). Nevertheless, performing the WAnT might be contraindicated during congested fixture periods or periods of intense training (Freitas et al., 2021). In such periods, additional exercise testing fatigue would be undesirable considering that WAnT might lead to blood lactate concentration higher than 11 mmol.L^–1^ in soccer players (Keir et al., 2013; Thom et al., 2020) and athletes of other sport (Jemni et al., 2006), and the use of surrogate measures of short-term muscle power might be an alternative.

The force–velocity (F-v) test, eliciting maximal power either in W (*P*max) or W.kg^–1^ (*rP*max), was also performed on a cycle ergometer; however, compared to the WAnT that used 30-s continuous exercise, it lasted a similar total duration, but included a series of sprints separated by 5 min of recovery (Vandewalle et al., 1985; Aloui et al., 2020). The intermittent protocol of the F-v test might explain the relatively low post-test blood lactate values reported in the literature, e.g., 3.5 mmol.L^–1^ (Sanchez et al., 2012), ∼6.5 mmol.L^–1^ (Bouhlel et al., 2010), and ∼7.5 mmol.L^–1^ (Blonc et al., 1998). In addition to *P*max and *rP*max, the F-v test provided two other indices, namely theoretical maximal force (F_0_) and velocity (v_0_) (Jaafar, 2017). Moreover, the F-v test has been used less often than WAnT in the evaluation of soccer players (Ben Ayed et al., 2011; Hammami et al., 2019). In this context, considering the importance of short-term muscle power for soccer performance, it would be interesting to examine the relationship of *P*peak and *rP*peak of the WAnT with indices of the F-v test.

Although the abovementioned studies enhanced our understanding of the metabolic demands of the WAnT and F-v test, little information has been available about their relationship, and particularly, about the possibility to predict Ppeak and rPpeak from F-v characteristics (i.e., *P*max, *rP*max, *F*_0_, and *v*_0_). Such information would be of great practical use for coaches and trainers working with soccer players to monitor performance, especially during periods of intense training and competition; if the prediction of Ppeak and rPpeak from F-v characteristics was possible, the F-v test as a less “lactic” exercise test, and consequently, inducing less fatigue, could be an alternative to the WAnT for the evaluation of *P*peak and *rP*peak (Bouhlel et al., 2010; Keir et al., 2013). Therefore, the aim of this work was to (a) develop a prediction equation of *P*peak and *rP*peak in soccer players, (b) examine the validity of this equation and its variation by performance level of rPpeak. The research hypothesis was that the development and validation of prediction equations would be possible considering the affinity of the WAnT and F-v test in terms of metabolic demands and mode of motion (Driss and Vandewalle, 2013).

## Materials and Methods

### Participants and Study Design

Participants were 158 adult men soccer players of soccer clubs of regional level (i.e., from the third, fourth, and fifth national league; sport experience 11.4 ± 4.5 years, mean ± standard deviation, approximately five weekly training units, age 22.6 ± 3.9 years, body mass 74.8 ± 7.8 kg, and height 178.3 ± 7.8 cm) who performed both WAnT and F-v test. An experimental (EXP, *n* = 79) and a control group (CON, *n* = 79) were used for the development and validation of prediction equation, respectively, of *P*peak and *rP*peak from F-v test. All participants provided informed consent after having been presented the benefits and risks of their participation in the present study.

### Equipment and Protocols

A weight scale (HD-351 Tanita, Illinois, United States) measured body mass (in the nearest 0.1 kg), a portable stadiometer (SECA, Leicester, United Kingdom) height (0.1 cm), and a caliper (Harpenden, West Sussex, United Kingdom) skinfolds (0.5 mm), respectively. Body mass index (BMI) was calculated as the quotient of body mass (kg) to height squared (m^2^). Body fat percentage (BF) was estimated from the sum of 10-skinfolds (cheek, wattle, chest I: pectoral, triceps, subscapular, abdominal, chest II: between the anterior axillary fold and the nipple, suprailiac, thigh and calf; BF = −41.32 + 12.59 × log_*e*_*x*, where *x* is the sum of 10 skinfolds) (Eston and Reilly, 2009). Fat mass and fat-free mass were calculated using the formulas “body mass × BF/100′”and “body mass – fat mass,” respectively. The F-v test was employed to assess *P*max, *rP*max, F_0_, and v_0_. This test employed various braking forces eliciting different pedaling velocities to evaluate the F-v relationship (Vandewalle et al., 1985; Aloui et al., 2020). The participants performed four sprints, each one lasting 7 s against incremental braking force (2, 3, 4, and 5 kg) on a cycle ergometer (Ergomedics 874, Monark, Sweden), interspersed by 5-min recovery periods. The WAnT was administered in the same ergometer as the F-v did (Dotan and Bar-Or, 1980; Miller, 2020). Briefly, participants were asked to pedal as fast as possible for 30 s against a braking force that was determined by the product of body mass in kilograms by 0.075. Both WAnT and F-v test have shown excellent intraclass correlation coefficient (>0.98) (Ingle and Tolfrey, 2013).

### Statistical and Data Analysis

A one-way analysis of variance examined differences in training, anthropometric, and physiological characteristics in the total sample of participants among quintiles of *rP*peak, i.e., low (*n* = 32; minimum, 8.96 W.kg^–1^ maximum, 10.60 W.kg^–1^), below average (*n* = 31; 10.61–11.18 W.kg^–1^), average (*n* = 32; 11.21–11.62 W.kg^–1^), above average (*n* = 31; 11.63–12.00 W.kg^–1^) and high *rP*peak (*n* = 32; 12.02–13.78 W.kg^–1^). The magnitude of these differences was evaluated by eta squared (η*^2^*). An independent *t*-test compared training, anthropometric, and physiological characteristics between EXP (*n* = 79) and CON (*n* = 79), and 95% confidence intervals were calculated for potential differences. The magnitude of differences between EXP and CON was assessed by Cohen’s *d*. In EXP, Pearson’s moment correlation coefficient (r) examined the relationship of *P*peak and *rP*peak with anthropometric and F-v characteristics (cut-off of *r*: < 0.10, trivial; 0.10–0.30, small; 0.30–0.50, moderate; 0.50–0.70, large; 0.70–0.90 very large; >0.90, perfect relationship). Also, in EXP, a stepwise regression analysis was carried out to identify predictors and develop prediction equations of *P*peak and *rP*peak. In CON, Bland–Altman plots examined the agreement between predicted and actual *P*peak and *rP*peak. All analyses were performed using GraphPad Prism v. 7.0 (GraphPad Software, San Diego, CA, United States) and IBM SPSS v.26.0 (SPSS, Chicago, IL, United States). Statistical significance for these analyses was set at alpha = 0.05.

## Results

Performance groups (quintiles of rPpeak) did not differ for training (*p* ≥ 0.282, *η^2^* ≤ 0.043) and anthropometric characteristics (*p* ≥ 0.133, *η^2^* ≤ 0.045) (Table 1). In addition to *rP*peak, they also differed in *P*peak, *P*mean, *rP*mean and FI of the WAnT, and *P*max, *rP*max and v0 of the F-v test (*p* ≤ 0.026, *η^2^* ≥ 0.071), but not for *F*_0_ (*p* = 0.800, *η^2^* = 0.011). Participants of high *rP*peak showed higher scores in the abovementioned variables than those with lower *rP*peak.

**TABLE 1:** Training, anthropometric and physiological characteristics in the total sample of participants (*n* = 158) and by quintiles of relative peak power of the Wingate anaerobic test.

Variable	Total (*n* = 158)	Low *rP*peak (*n* = 32)	Below average *rP*peak (*n* = 31)	Average *rP*peak (*n* = 32)	Above average *rP*peak (*n* = 31)	High *rP*peak (*n* = 32)	*P*	*η ^2^*
**Training and anthropometry**								
Sport experience (years)	11.4 ± 4.5	12.5 ± 5.1	10.3 ± 4.7	11.3 ± 4.5	10.3 ± 4.5	12.1 ± 3.8	0.439	0.038
T.U. (number.wk^–1^)	4.8 ± 1.4	4.5 ± 1.1	5.2 ± 1.6	5.1 ± 1.2	4.6 ± 1.3	4.5 ± 1.7	0.282	0.043
Duration of T.U. (min)	91.3 ± 19.6	92.9 ± 12.2	90.0 ± 12.2	91.7 ± 20.1	94.4 ± 21.0	87.9 ± 26.0	0.800	0.014
Duration of training (min.wk^–1^)	454.5 ± 179.7	429.3 ± 128.3	471.7 ± 157.4	478.1 ± 176.3	453.4 ± 183.6	439.4 ± 230.5	0.303	0.011
Age (years)	22.6 ± 3.9	23.8 ± 4.6	21.3 ± 3.0	22.9 ± 4.2	22.4 ± 3.5	22.3 ± 3.4	0.133	0.045
Height (cm)	178.3 ± 7.8	176.6 ± 6.3	178.5 ± 5.8	179.7 ± 5.3	178.4 ± 6.3	178.3 ± 5.6	0.344	0.029
Body mass (kg)	74.8 ± 7.8	74.7 ± 11.2	76.2 ± 7.7	75.1 ± 6.7	74.3 ± 7.2	73.5 ± 5.1	0.738	0.013
BMI (kg.m^–2^)	23.5 ± 1.9	23.9 ± 2.8	23.9 ± 1.6	23.2 ± 1.7	23.3 ± 1.4	23.1 ± 1.5	0.296	0.031
BF (%)	15.8 ± 3.3	16.9 ± 4.1	16.2 ± 3.3	15.6 ± 3.0	15.0 ± 3.3	15.2 ± 2.3	0.153	0.043
**Force-velocity test**								
*P*max (W)	1129 ± 222	1017 ± 230	1105 ± 165	1129 ± 171	1187 ± 267	1207 ± 220	0.005	0.093
*rP*max (W.kg^–1^)	15.12 ± 2.66	13.66 ± 2.50	14.51 ± 1.74	15.05 ± 2.00	16.01 ± 3.34	16.38 ± 2.61	<0.001	0.140
v_0_ (rpm)	220.3 ± 18.9	209.1 ± 22.4	213.9 ± 18.9	221.8 ± 15.2	224.8 ± 15.3	232.1 ± 12.8	<0.001	0.186
F_0_ (kg)	20.74 ± 4.94	19.88 ± 5.89	20.89 ± 3.97	20.57 ± 4.00	21.42 ± 6.04	20.95 ± 4.58	0.800	0.011
**Wingate anaerobic test**								
Ppeak (W)	846.8 ± 101.9	752.4 ± 110.2	828.5 ± 87.4	862.2 ± 75.0	876.3 ± 85.4	914.7 ± 70.7	<0.001	0.293
rPpeak (W.kg^–1^)	11.33 ± 0.85	10.08 ± 0.41	10.91 ± 0.18	11.41 ± 0.12	11.80 ± 0.09	12.43 ± 0.36	<0.001	0.904
Pmean (W)	656.3 ± 72.7	596.6 ± 80.7	645.8 ± 60.7	669.4 ± 53.5	677.02 ± 72.5	693.0 ± 55.9	<0.001	0.214
rPmean (W.kg^–1^)	8.81 ± 0.78	8.02 ± 0.72	8.50 ± 0.61	8.95 ± 0.74	9.15 ± 0.44	9.44 ± 0.44	<0.001	0.409
FI (%)	43.29 ± 7.62	40.58 ± 9.47	42.35 ± 7.94	42.60 ± 7.80	44.49 ± 6.65	46.50 ± 4.34	0.026	0.071

*Values were presented as mean ± standard deviation (SD). T.U. = training units; BMI = body mass index, BF = body fat percentage, Pmax = absolute maximal power, rPmax = relative maximal power, v_0_ = theoretical maximal velocity, F_0_ = theoretical maximal force, Ppeak = absolute peak power, rPpeak = relative peak power, Pmean = absolute mean power, rPmean = relative mean power, FI = fatigue index.*

**FIGURE 1:**
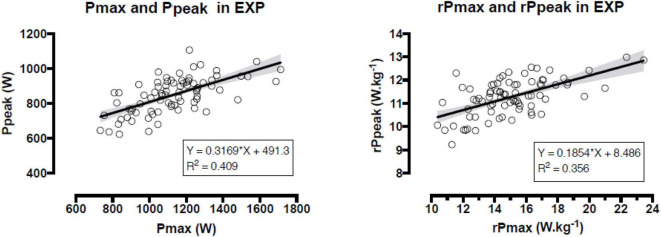
Relationship between maximal power of the force-velocity test and peak power of the Wingate anaerobic test (both in absolute and relative to body mass values) in the experimental group. *P*max = absolute maximal power, *rP*max = relative maximal power, *P*peak = absolute peak power, *rP*peak = relative peak power.

In EXP, Ppeak correlated very largely with body mass (*r* = 0.787), fat-free mass (*r* = 0.782), largely with *P*max (*r* = 0.639), BMI (*r* = 0.603), fat mass (*r* = 0.611) height (*r* = 0.558), moderately with F_0_ (*r* = 0.481), BF (*r* = 0.471; *p* < 0.001), but not with *rP*max (*r* = 0.190, *p* = 0.093) age (*r* = 0.189, *p* = 0.096), and v_0_ (*r* = 0.128, *p* = 0.262); *rP*peak correlated with *rP*max (largely; *r* = 0.596, *p* < 0.001), v_0_ (moderately; *r* = 0.341, *p* = 0.002), F_0_ (small magnitude; *r* = 0.280, *p* = 0.012), BF (*r* = −0.230, *p* = 0.042), fat mass (*r* = −0.242, *p* = 0.032). The correlations between Ppeak and *P*max, rPpeak and *rP*max in EXP are presented in Figure 1.

Compared with CON, EXP had similar sport experience (mean difference −0.6 years; 95% CI, −2.4, 1.2), weekly training units (−0.4; 95% CI, −0.9, 0.1), duration of training unit (2.9 min; 95% CI, −4.2, 10.1), weekly duration of training (−22.8 min; 95% CI, −88.6, 42.9), age (−0.6 years; 95% CI, −1.8, 0.6), height (−1.2 cm; 95% CI, −3.0, 0.6) and body mass (1.0 kg; 95% CI, −1.5, 3.5), but higher BMI (0.6 kg.m^–2^; 95% CI, 0, 1.2) and BF (1.2%; 95% CI, 0.2, 2.2) (Table 2). With regards to anaerobic characteristics, no difference was observed in *P*max (−3W; 95% CI, −73, 66), *rP*max (−0.17 W.kg^–1^; 95% CI, −1.01, 0.67), v_0_ (−1.7 rpm; 95% CI, −7.6, 4.3), F_0_ (0.01 kg; 95% CI, −1.55, 1.56), Ppeak (3.4 W; 95% CI, −28.8, 35.5), rPpeak (−0.09 W.kg^–1^; 95% CI, −0.36, 0.17), Pmean (−14.7 W; 95% CI, −37.8, 8.3) and FI (2.36%; 95% CI, −0.4, 4.75), whereas EXP had lower rPmean than CON (−0.34 W.kg^–1^; 95% CI, −0.61, −0.07).

**TABLE 2:** Training, anthropometric, and anaerobic characteristics in the experimental and control group.

Variable	CON (*n* = 79)	EXP (*n* = 79)	*p*	*d*
Experience (years)	11.7 ± 4.6	11.1 ± 4.5	0.484	0.132
T.U. (number.wk^–1^)	5.0 ± 1.4	4.6 ± 1.4	0.113	0.286
Duration of T.U. (min)	89.7 ± 20.7	92.7 ± 18.5	0.418	0.153
Duration of training (min.wk^–1^)	466.3 ± 183.1	443.5 ± 177.3	0.493	0.016
Age (years)	22.9 ± 3.9	22.3 ± 3.8	0.313	0.156
Height (cm)	178.9 ± 5.7	177.7 ± 6.1	0.186	0.203
Body mass (kg)	74.3 ± 6.9	75.3 ± 8.6	0.424	0.128
BMI (kg.m^–2^)	23.2 ± 1.6	23.8 ± 2.0	0.035	0.331
BF (%)	15.2 ± 2.8	16.4 ± 3.7	0.024	0.366
Pmax (W)	1130 ± 238	1127 ± 206	0.922	0.013
rPmax (W.kg^–1^)	15.20 ± 2.80	15.03 ± 2.53	0.686	0.064
v_0_ (rpm)	221.2 ± 20.2	219.5 ± 17.5	0.578	0.090
F_0_ (kg)	20.73 ± 5.45	20.74 ± 4.41	0.994	0.002
Ppeak (W)	845.1 ± 102.3	848.4 ± 102.1	0.837	0.032
rPpeak (W.kg^–1^)	11.38 ± 0.90	11.29 ± 0.78	0.489	0.107
Pmean (W)	663.6 ± 78.5	648.9 ± 66.0	0.208	0.203
rPmean (W.kg^–1^)	8.95 ± 0.79	8.68 ± 0.75	0.031	0.351
FI (%)	42.12 ± 7.39	44.48 ± 7.72	0.054	0.312

*Values were presented as mean ± standard deviation (SD). T.U. = training units; BMI = body mass index, BF = body fat percentage, Pmax = absolute maximal power, rPmax = relative maximal power, v_0_ = theoretical maximal velocity, F_0_ = theoretical maximal force, Ppeak = absolute peak power, rPpeak = relative peak power, Pmean = absolute mean power, rPmean = relative mean power, FI = fatigue index.*

**FIGURE 2:**
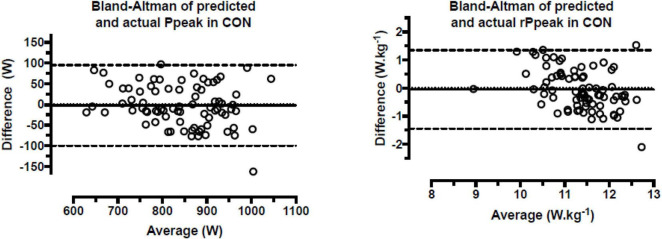
Bland–Altman plots of predicted and actual peak power of the control group in the Wingate anaerobic test. *P*peak = absolute peak power; *rP*peak = relative peak power; CON = control group; Difference = predicted – actual; Average = (predicted + actual)/2; dashed lines represent 95% confidence intervals of bias.

Ppeak in EXP could be predicted from *P*max, body mass and F_0_, and rPpeak from *rP*max and v_0_ using the formulas presented in Table 3. Applying these formulas in CON, no bias was observed between actual and predicted Ppeak in W (mean difference 2.5 ± 49.8 W; 95% CI, −8.7, 13.6; *p* = 0.661) and W.kg^–1^ (mean difference 0.05 ± 0.71 W.kg^–1^; 95% CI, −0.11, 0.21, *p* = 0.525) (Figure 2).

**TABLE 3:** Summary of regression analysis in the experimental group (*n* = 79).

Dependent variable	Formula	*R*	*R* ^2^	SEE
*P*peak (W)	44.251 + 7.431 × body mass (kg) + 0.576 × *P*max (W) – 19.512 × F_0_ (kg)	0.912	0.833	42.616
*rP*peak (W.kg^–1^)	3.148 + 0.218 × *rP*max (W.kg^–1^) + *v*_0_ (rpm)	0.765	0.585	0.514

*R = correlation coefficient, R^2^ = coefficient of determination, SEE = standard error of the estimate, Pmax = absolute maximal power, rPmax = relative maximal power, v_0_ = theoretical maximal velocity, F_0_ = theoretical maximal force, Ppeak = absolute peak power, rPpeak = relative peak power.*

## Discussion

The main findings of the present study were that (a) *P*peak and *rP*peak correlated with *P*max and *rP*max, respectively, (b) the best correlates of *P*peak and *rP*peak were body mass and v_0_, respectively, (c) *P*peak could be predicted from *P*max, body mass and F_0_, (d) *rP*peak could be predicted from *rP*max and v_0_, and (e) no bias was observed between actual and predicted Ppeak and *rP*peak.

The large correlation between *P*peak and *P*max, and rPpeak and *rP*max highlighted the possibility to use WAnT and F-v test interchangeably considering the short-term duration and need for maximal effort of both tests (Driss and Vandewalle, 2013). In addition, the larger values of *P*max and *rP*max than *P*peak and *rP*peak were in agreement with previous studies using both the tests (Jemni et al., 2006; Souissi et al., 2008; Ingle and Tolfrey, 2013). An explanation of this difference might be that the highest power output in the F-v test was estimated, whereas it was measured in the WAnT at a given braking force (which was set considering each participant’s body mass).

Comparing the predictors of *P*peak and *rP*peak, it was observed that different anthropometric and F-v characteristics played a predicting role in each case. The best predictor of *P*peak was body mass highlighting the relationship of muscle power with human size (Bahenský et al., 2020; Taketomi et al., 2021). Previously, it was shown that *rP*peak was related with 5 m, 30 m sprint times, maximal voluntary isometric contraction of the knee extensors, half squat repetition maximal and countermovement jump height in soccer players (Boraczyński et al., 2020), performance of short and maximal effort that might be evaluated by the F-v test. Comparing the prediction models of *P*peak and *rP*eak, it was observed that the coefficient of determination was higher for the absolute than for the relatively score of peak power. Peak power related very largely with body mass, and consequently, since body mass was partitioned out in *rP*peak, a weaker model was shown for the relative score of peak power.

A limitation of this study was that the findings referred to specific performances in the selected anaerobic tests. Since the bias was larger in low and high anaerobic performances, caution would be needed to generalize the developed prediction equations to other populations. However, there was large interindividual variability in the agreement between the actual and the predicted scores; thus, the use of the developed equations to predict *P*peak and *rP*peak should be avoided when precision at an individual level would be needed in soccer players with small interindividual variation. On the other hand, the developed equations provided a practical tool to coaches and trainers to predict the average *P*peak and *rP*peak of a group of athletes from their F-v test. Accordingly, the F-v test might be considered as a diagnostic tool of team instead of individual WAnT performance. Future studies should examine the interchangeability of these tests in soccer players differing for sex, age, and performance level.

## Conclusion

The findings of this work provided indirect estimates of the average *P*peak and *rP*peak of the WAnT for a group of players that would be useful especially in periods when this test should not be applied considering the fatigue it causes. In this context, the F-v test can be considered as an alternative of exercise testing for the average *P*peak and *rP*peak of a group of soccer players rather than for predicting individual scores when the interindividual variation of performance is small.

## Data Availability Statement

The raw data supporting the conclusions of this article will be made available by the authors, without undue reservation.

## Ethics Statement

The studies involving human participants were reviewed and approved by the Institutional Review Board of EPL. The study was conducted according to the guidelines of the Declaration of Helsinki. The patients/participants provided their written informed consent to participate in this study.

## Author Contributions

PN collected all data, performed the analyses, and drafted the manuscript. BK helped in drafting the manuscript. Both authors contributed to the article and approved the submitted version.

## Conflict of Interest

The authors declare that the research was conducted in the absence of any commercial or financial relationships that could be construed as a potential conflict of interest.

## Publisher’s Note

All claims expressed in this article are solely those of the authors and do not necessarily represent those of their affiliated organizations, or those of the publisher, the editors and the reviewers. Any product that may be evaluated in this article, or claim that may be made by its manufacturer, is not guaranteed or endorsed by the publisher.
